# Physicochemical data of *p*-cresol, butyric acid, and ammonia

**DOI:** 10.1016/j.dib.2019.104356

**Published:** 2019-08-22

**Authors:** Praveen Kolar, John Classen, Steven G. Hall

**Affiliations:** North Carolina State University, Biological and Agricultural Engineering, 278 Weaver Labs, Campus Box 7625, Raleigh, USA, NC 27695-7625

**Keywords:** Butyric acid, *p*-cresol, Ammonia, Indole, Density functional theory

## Abstract

There is a renewed interest in treating odorous contaminants such as butyric acid, *p*-cresol, and ammonia that are emitted from animal farming operations. However, developing newer treatment technologies require quantitative information regarding the properties of the target pollutants. Therefore, in this communication, baseline data related to physicochemical and thermodynamic properties of butyric acid, *p-*cresol, and ammonia were predicted using computational chemistry. Density functional theory was employed via B3LYP functional coupled with polarized 6-31G (d) basis set without any solvent effects using Gaussian 16W and GaussView6. The predicted baseline properties collected here are expected to be useful to scientists and engineers working in environmental mitigation technologies in developing treatment processes and make the animal agricultural industry environmental friendly and sustainable.

**Table:** Specifications Table

Subject area	Agriculture and Environmental Engineering.
More specific subject area	Odor mitigation.
Type of data	Tables, plots, images, and text output files.
How data was acquired	Gaussian 16W and GaussView 6 by Gaussian Inc.
Data format	Software code, Raw data, processed and plotted data, and images.
Experimental factors	The simulations were performed using Gaussian 16W using density functional theory and the acquired data were analyzed using GaussView 6 to extract molecular electrostatic potential maps (ESP), natural bond orbitals (NBO), frontier orbital diagrams, Mulliken, atomic polar tensor (APT), Merz-Kollman and Hirshfeld charges.
Parameters for data collection	All simulations were performed at 298 K and 1 atm in the gas phase without the presence of any solvent. All software codes, checkpoint files, and log files are attached as text files.
Description of data collection	The physical and chemical properties data were obtained using via B3LYP functional coupled with polarized 6-31G (d) basis set.
Data source location	City/Town/Region: Raleigh, North Carolina. Country: USA.
Data accessibility	All data associated with this article are hosted with the article.

**Table:** 

**Value of the data** • There is a significant interest in mitigating *p*-cresol, butyric acid, and ammonia from animal farming operations. • Design of treatment processes require data on physical, chemical, and thermodynamic properties. • Density functional theory was employed to predict the solubility, entropy, molecular structure, chemical hardness, reactivity, and other properties that are not readily available. • Researchers across the world can use these data to design and develop improved waste treatment processes. • The data presented can be used to predict the electrophilic, nucleophilic regions, and reactive sites in the molecules studied. • The raw data may also be used to obtain additional information such as bond lengths, bond angles, and spectroscopic and vibrational properties.

## 1 Data

Table 1 summarizes the energy, dipole, and polarizability, heat capacity, and standard molar entropy of the selected compounds obtained from the DFT computation. The relatively large dipole moments suggested that all three compounds were non-polar and the order of the polarity was found to be ammonia > butyric acid > *p*-cresol. In addition, as expected, the polarizability and standard molar entropy were found to increase with the size of the molecule.

**Table 1:** Thermodynamic properties of butyric acid, *p-*cresol, and ammonia molecule.

Compound	Energy (Hartree) (kJ mol^−1^)	Dipole (Debye)	Polarizability (C. m^2^ V^−1^)	Heat Capacity (Cal mol^−1^ K^−1^)	Entropy (Cal mol^−1^ K^−1^)
Ammonia	−56.510 −148,367.0	1.91	8.3	6.28	45.98
*p*-cresol	−346.642 −910,108.6	1.32	71.96	27.86	87.84
Butyric acid	−307.582 −807,556.5	1.73	47.77	23.68	83.78

Energy = Electronic + Thermal energy; Hartree = 2625.5 kJ mol^−1^; Debye = 3.36 × 10^−30^ C m.

**Fig. 1:**
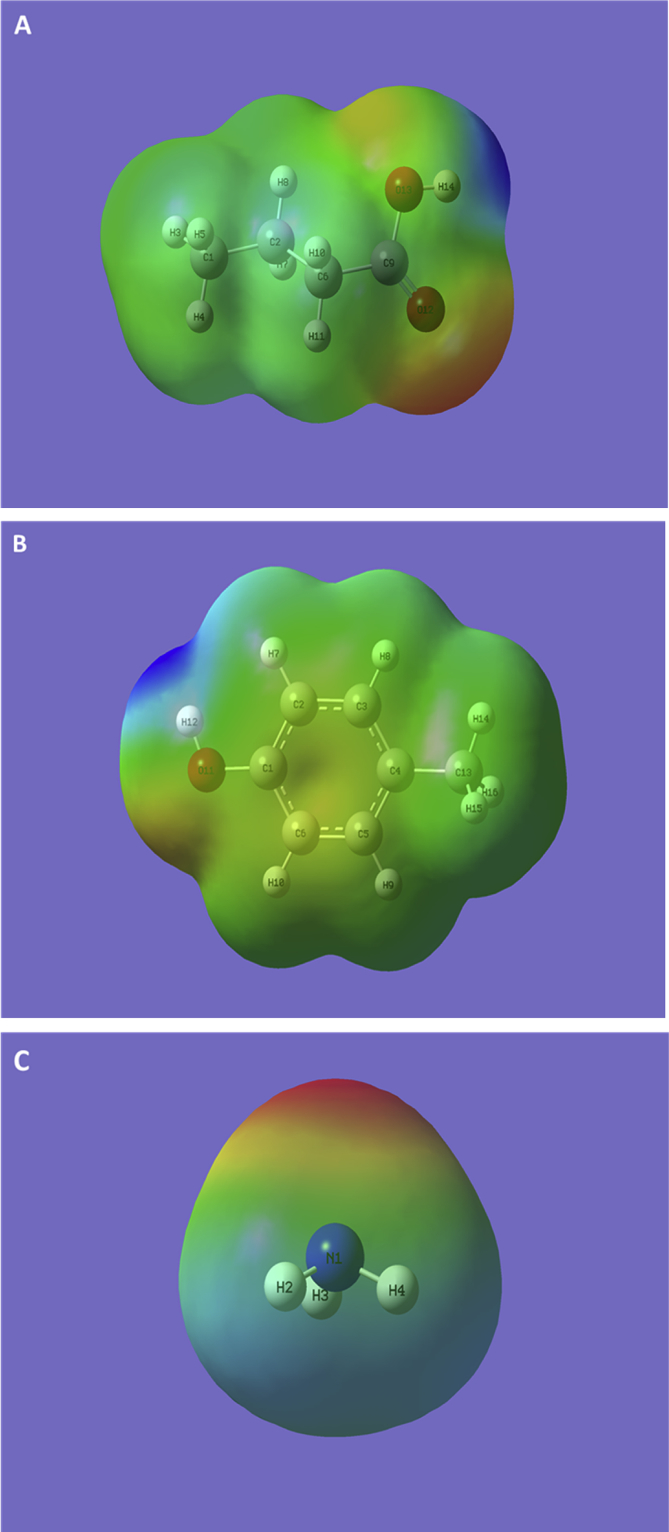
The molecular electrostatic potential (ESP) maps of butyric acid (A), *p*-cresol (B), and ammonia (C) molecule.

The molecular electrostatic potential maps (ESP) reveal electron-rich and electron-poor areas in a molecule, which may be used to visually analyze and predict the reactivity of a given molecule based on color-red being most negative and blue being the most positive [1], [2]. As can be seen from butyric acid's ESP (Fig. 1), the positive-electrophilic center is located around H14 and the region around O12 indicated negative-nucleophilic region, due to the presence of Π bonds. Similarly, for *p*-cresol, the region around H12 exhibited most (positive) attraction while the region around O11 indicated most (negative) repulsion. For ammonia, as expected, the region around N1 was found to be the nucleophilic center while the region around H2, H3, and H4 indicated electrophilic zone.

**Fig. 2:**
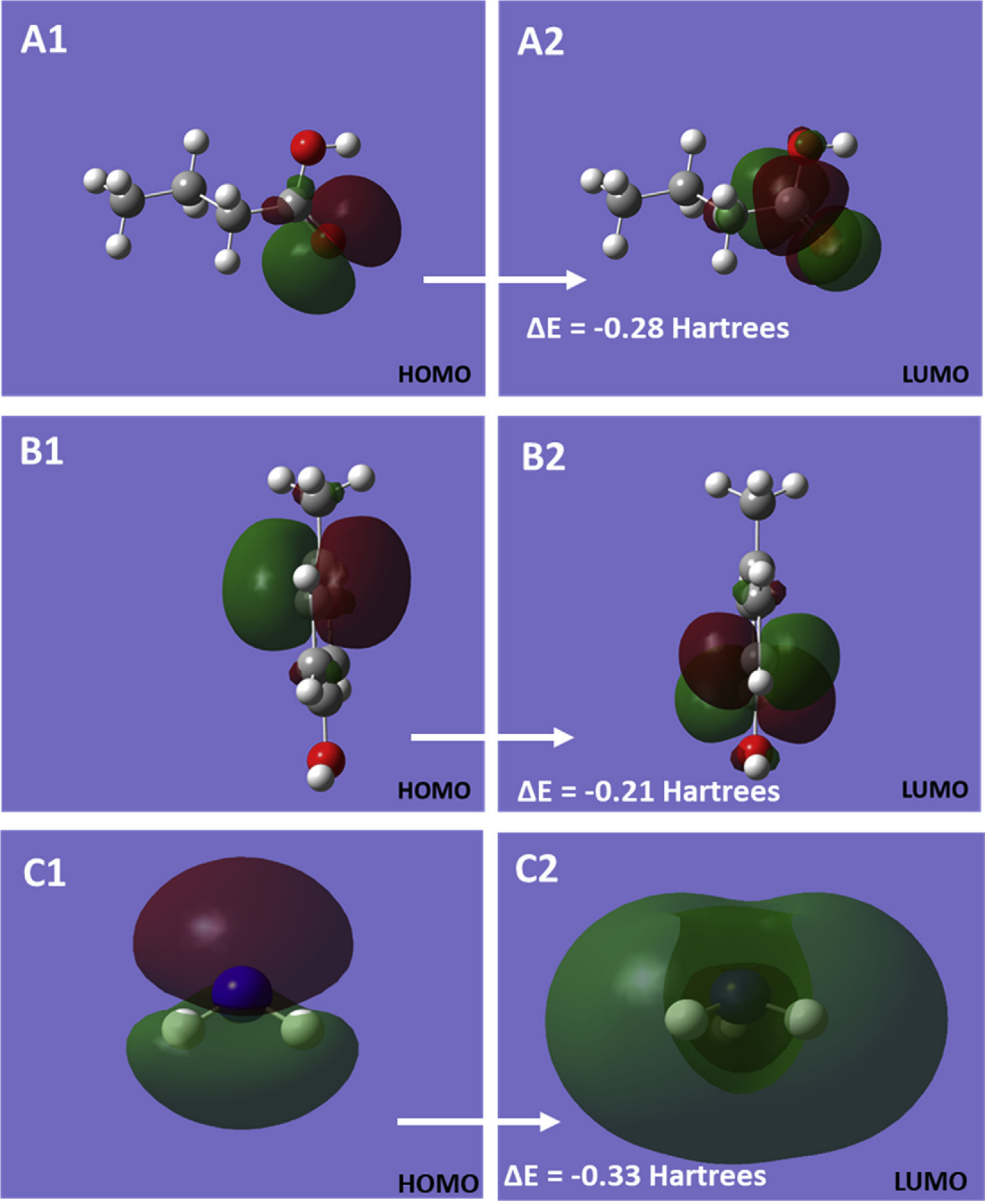
The frontier orbitals of butyric acid (A), *p*-cresol (B), and ammonia (C) molecule.

The collected data was extracted to plot the highest occupied molecular orbital (HOMO) and lowest unoccupied molecular orbital (LUMO) for butyric acid, *p*-cresol, and ammonia (Fig. 2). As suggested by that [3], HOMO and LUMO indicate the propensity of electron donation and acceptance of the orbital and therefore the energy band gap provides information about the molecular stability [4]. Based on the DFT calculations, the HOMO-LUMO band gaps for butyric acid, *p*-cresol, and ammonia were estimated −0.28, −0.21, and −0.33 Hartrees, respectively. In addition, the physicochemical properties were estimated using the HOMO and LUMO energies as per [5] and presented in Table 2.

**Table 2:** Predicted chemical properties of butyric acid, *p*-cresol, and ammonia molecule.

Compound	Chemical Potential (μ) (Hartrees)	Chemical hardness (η) (Hartrees)	Global Electrophilicity index (ω) (Hartrees)	Electronegativity (χ) (Hartrees)	Chemical Softness (S) (Hartrees)
Butyric acid	−0.130	0.140	0.06	0.130	7.13
*p*-cresol	−0.104	0.106	0.05	0.104	9.36
Ammonia	−0.087	0.165	0.02	0.087	6.04

The chemical hardness data indicated that *p*-cresol molecule has more propensity to exchange electrons with other molecules and therefore was the least stable when compared to butyric acid and ammonia. Further, as proposed by Ref. [5], the values of global electrophilicity indices (ω) may be used to predict the reactivities of molecules in polar media.

The charge transfer within each molecule and the intramolecular bonding was studied by comparing the stabilization energies E^(2)^ estimated via the second order perturbation theory analysis of Fock Matrix in the NBO analyses as described by Ref. [6]. Considering that stabilization energy is an indicator of the extent of interaction between Lewis donor and non-Lewis acceptor orbitals, for butyric acid molecule, the O13 atom served as an electron donor while the antibonding orbital of C9–O12 acted as an electron acceptor with stabilization energy of 46 kcal mol^−1^. Similarly, for *p-*cresol molecule, the O11 atom, due to the presence of a lone pair was able to donate electrons to antibonding C1–C2 orbital resulting in stabilization energy of 28 kcal mol^−1^. Further, the antibonding C1–C2 orbital conjugated with antibonding C3–C4 orbital with significantly higher energy (E^(2)^ = 215 kcal mol^−1^). However, for the ammonia molecule, all interaction energies were below 0.5 kcal mol^−1^.

**Fig. 3:**
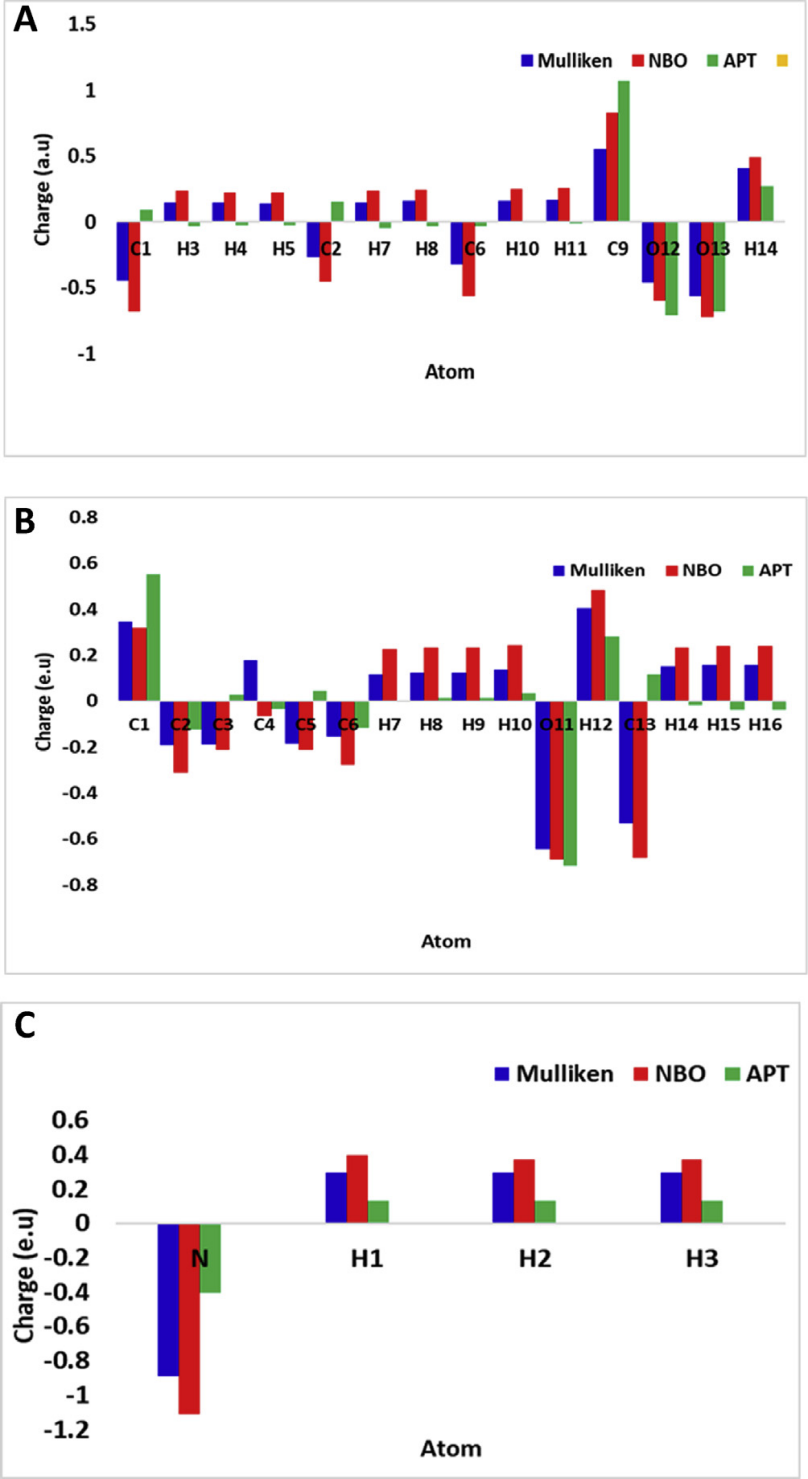
Atomic charge distributions of butyric acid (A), *p*-cresol (B), and ammonia (C) molecule via Mulliken, natural bond orbitals (NBO), and atomic polar tensor (APT) calculations.

Atomic charge distribution analysis provides information about the extent of individual net charges exhibited by the atoms. The data for butyric acid, *p*-cresol, and ammonia molecules obtained by Mulliken, natural bond orbitals (NBO), and atomic polar tensor (APT) calculations are depicted in Fig. 3. From the model's population analysis, it appears that O12 and O13 in butyric acid, O11 and C13 in *p*-cresol, N in ammonia are relatively more active than other atoms and therefore can act as active sites in chemical reactions. Additionally, atomic charges computed via Merz-Kollman and Hirshfeld approaches are available in the output text file (Supplementary files) for further analyses, if desired.

## 2 Materials and methods

All computations were performed in commercially procured Gaussian 16W [7] interfaced with GaussView6 [8] developed by Gaussian Inc., Wallingford, CT, USA. Each molecule was subjected to ground state optimization followed by frequency calculations (Opt + Freq) via DFT using B3LYP functional coupled with polarized 6-31G (d) basis set at 298 K and 1 atm in the gas phase (without the presence of any solvent around the target molecules). The inputs provided were (1) unoptimized chemical structure, (2) the choice of job type (Opt + Freq), (3) method type (ground state), (4) the model type (DFT), (5) the density functional (B3YLP), and (4) the basis set (6-31G (d)). All software input codes are provided in the supplementary information. The output (after the computations) were obtained as check point files and log files, which were interfaced with GaussView6 to extract and view the optimized geometry and data associated with electronic energies, dipoles, and polarizabilities (Table 1) while the checkpoint files were used prepare molecular electrostatic potential maps (Fig. 1), frontier orbital diagrams (Fig. 2), and analyze the atomic charges (Fig. 3). Further, the physicochemical properties were estimated from the HOMO and LUMO energies by substituting –E(HOMO) and -E(LUMO) for ionization potential (IP) and electron affinity (EA) [6] and using the mathematical relationships relations, Chemical Potential (μ) = −0.5 (IP + EA) = -Electronegativity (Χ), Chemical Hardness (η) = 0.5 (IP-EA), and Global Electrophilicity Index (ω) = μˆ2/2η, and Global Softness (S) = 1/ω [9] (Table 2). All code (SI-1 A-C), check point (SI-2 A-C) and log (SI-3 A-C), files generated as a part of computation are presented in the supplementary information.
